# Comparison of External Beam Radiation Therapy Modalities for Hepatocellular Carcinoma With Macrovascular Invasion: A Meta-Analysis and Systematic Review

**DOI:** 10.3389/fonc.2022.829708

**Published:** 2022-02-15

**Authors:** Guanheng Wu, Guomin Huang, Jianwen Huang, Ligong Lu, Shaojun Peng, Yong Li, Wei Zhao

**Affiliations:** Zhuhai Precision Medical Center, Guangdong Provincial Key Laboratory of Tumor Interventional Diagnosis and Treatment, Zhuhai People’s Hospital, Zhuhai Hospital Affiliated with Jinan University, Jinan University, Zhuhai, China

**Keywords:** radiation therapy, hepatocellular carcinoma, macrovascular invasion, portal vein tumor thrombosis, conformal radiation therapy, stereotactic body radiotherapy, particle therapy

## Abstract

**Purpose:**

We performed a systematic review and meta-analysis to compare external beam radiation therapy modalities for hepatocellular carcinoma (HCC) with macrovascular invasion (MVI).

**Methods:**

Studies were selected from online databases from the date of inception to November 2021. The outcomes of interest were overall survival (OS), objective response rate (ORR), and local control rate (LCR).

**Results:**

Forty-four studies (n = 3730) were selected from 1050 articles. The pooled 1-year OS were 60.9%, 45.3%, and 44.9 for particle radiotherapy (PRT) group, conventional radiotherapy (CRT), and stereotactic body radiotherapy (SBRT) group, respectively; *p* = 0.005 and 0.002 for PRT vs. CRT and SBRT, respectively. Both the PRT group and the SBRT group have the advantage over the CRT group in the pooled ORR. The PRT group showed significantly higher than the CRT group (*p* = 0.007) in LCR. For combination therapy, CRT plus transarterial chemoembolization can prolong survival than CRT alone (*p* = 0.006 for 1-year OS; *p* = 0.014 for 2-year OS). Among grade ≥ 3 complications, the most frequent type of toxicity in CRT, SBRT, PRT group was hematological toxicity, hepatotoxicity, dermatological toxicity, respectively.

**Conclusions:**

Among patients with HCC with MVI, the 1-year OS and the 2-year OS were both higher in the PRT group than in the CRT, SBRT groups. The ORR was similar between the PRT and SBRT groups. The combination therapy based on radiotherapy is expectable. PRT is associated with less complications than photon radiotherapy.

## Introduction

According to the Global Cancer Statistics 2020, primary liver cancer is the sixth most common malignancy and the third leading cause of cancer-related death worldwide, with around 906,000 new cases and 830,000 deaths reported in 2020. Approximately 80% of these cases were hepatocellular carcinomas (HCCs) (1). As the clinical manifestations are not evident, most cases of HCCs only detected at the advanced stage. Microvascular invasion (MVI) is common in HCC. Portal vein tumor thrombus (PVTT) occurs in 10–40% of patients with HCC (2, 3). The median survival time is significantly lower in patients with PVTT than in those without (4). Worse outcomes are noted when inferior vena cava thrombi are present (5). There are several treatments for HCC, such as transarterial chemoembolization (TACE), hepatic arterial infusion chemotherapy (HAIC), percutaneous ethanol injection (PEIT), and radiofrequency ablation (RFA) (6). However, a tumor thrombus alters the blood supply route to the liver, reduces nutrient supplement, and further reduces the liver function reserve. Therefore, most treatments are no longer effective. Sorafenib is one of the preferred treatments of choice for this condition (6). However, the overall response rate of HCC with MVI to sorafenib is low, and the associated toxicity is severe (7, 8). It is therefore important to consider other effective treatments.

External beam radiation therapy (EBRT) is one of the promising treatments. Previously, the tolerated liver dose was considered to be lower than the tumor killing dose, and therefore, this treatment could not be used for liver cancer (9, 10). However, in recent years, imaging and dose control techniques have made great progress, with reduced toxicity to normal liver tissue. A meta- analysis showed that the 1-year overall survival (OS) and response rate for stereotactic body radiation therapy (SBRT) were 43.8% and 70.7% %, respectively (11). These data objectively reflect the therapeutic advantage of EBRT for HCC with MVI. Recently, several high-quality studies have reported the advantages of EBRT for unresectable HCC, especially for particle radiotherapy (PRT), which shows the preponderance of high response rate, high control and low toxicity (12). However, due to the lack of PRT centers, it is difficult to conduct a head-to-head comparison study with a large sample size for PRT versus other EBRTs for HCC with MVI. Therefore, we conducted this meta-analysis to compare the safety and effectiveness of PRT and photon therapy for HCC with MVI. Meanwhile, it serves to update findings related to EBRT from a previous meta-analysis (11).

## Methods

### Search and Selection Criteria

This study followed the Preferred Reporting Items for Systematic Reviews and Meta-analyses (PRISMA) reporting guidelines (13). The protocol we designed defined inclusion criteria, search strategy, outcomes of interest, and analysis plan.

We searched Medline (Ovid), Embase, Clinicaltrials, Web of Science, the Cochrane Central Register of Controlled Trials, and the Cochrane Database of Systematic Reviews, from the date of inception of each database to November 2021. The following keywords or terms were used: “(hepatocellular carcinoma) OR (HCC) OR (hepatoma)” AND “(external beam radiation therapy) OR (stereotactic body radiation therapy) OR (conformal radiotherapy) OR (particle radiotherapy)” AND “(thrombosis)”. Additional references were acquired through manual searches of the reference lists. No filters were used, but only papers written in English were included.

The cohorts in the studies had to meet criteria for inclusion as follows: 1) HCC with macrovascular invasion; 2) treatment with EBRT; 3) reported outcomes of interest (i.e., overall survival, response rate, and adverse events). We excluded case reports with fewer than fifteen patients, reviews, letters, and editorial comments. If more than one available study was conducted from the same treatment center in overlapping timeframes, the study with the biggest group and/or highest quality of article was selected. HCC with microvascular invasion was excluded. The conventional radiotherapy (CRT) included three dimensional conformal radiation therapy (3D-CRT), image-guide radiotherapy (IGRT), intensity-modulated radiotherapy (IMRT). SBRT and CRT are difference type of photon therapies. PRT usually means radiotherapy using beams of protons, carbon ions, or other charged particles. Hematological toxicity includes leukopenia, anemia, thrombocytopenia, etc. Hepatotoxicity includes increased ALT, AST, ALP, bilirubin, GGT level, hypoproteinemia, etc. Dermatological toxicity refers to skin reactions. Gastrointestinal toxicity includes nausea, vomit, anorexia, diarrhea, etc. Objective response rate (ORR) was defined as complete response (CR) plus partial response (PR). Local control rate (LCR) means ORR plus stable disease (SD).

### Data Extraction

The details were extracted in a standardized pilot-tested form by two reviewers independently. A third investigator reviewed all data entries. The lists we extracted as follows: study design, country, study period, number of patients, patients’ characteristics (percentage of male patients, age, diameter of lesion, Child-Pugh Class, previous treatment), interventions (radiation dose, modality for EBRT), length of follow-up, median overall survival, and outcomes of interest.

### Statistical Analysis

We prespecified the analysis plan for this protocol. We transformed the rates using the variance stabilizing double arcsine transformation. Then, we pooled the transformation rates with random-effect models and assessed heterogeneity. Heterogeneity among studies was tested using Cochran’s Q and the I² statistic. I² values greater than 50% indicating high heterogeneity. Q-test was use in comparisons among groups (11). We performed a subgroup analysis and pooled the rates of interest outcomes for the different types of EBRT. Egger’s test was used to detect publication bias. When textual information in the included study was insufficient, two reviewers independently collected the information from the graphs using Engauge Digitizer 11.1. P < 0.5 was considered as statistical significance. All statistical analyses were conducted using STATA, version 15.1 (Stata Corporation, College Station, TX, USA).

### Assessment of Study Quality

Because most of the studies included in our systematic review and meta-analysis were non-comparative studies, we used the modified Newcastle-Ottawa quality assessment scale. The evaluation of quality was independently conducted by two investigators. Any disagreements were resolved by a third investigator.

## Results

### Study Selection and Quality Assessment

**Figure 1:**
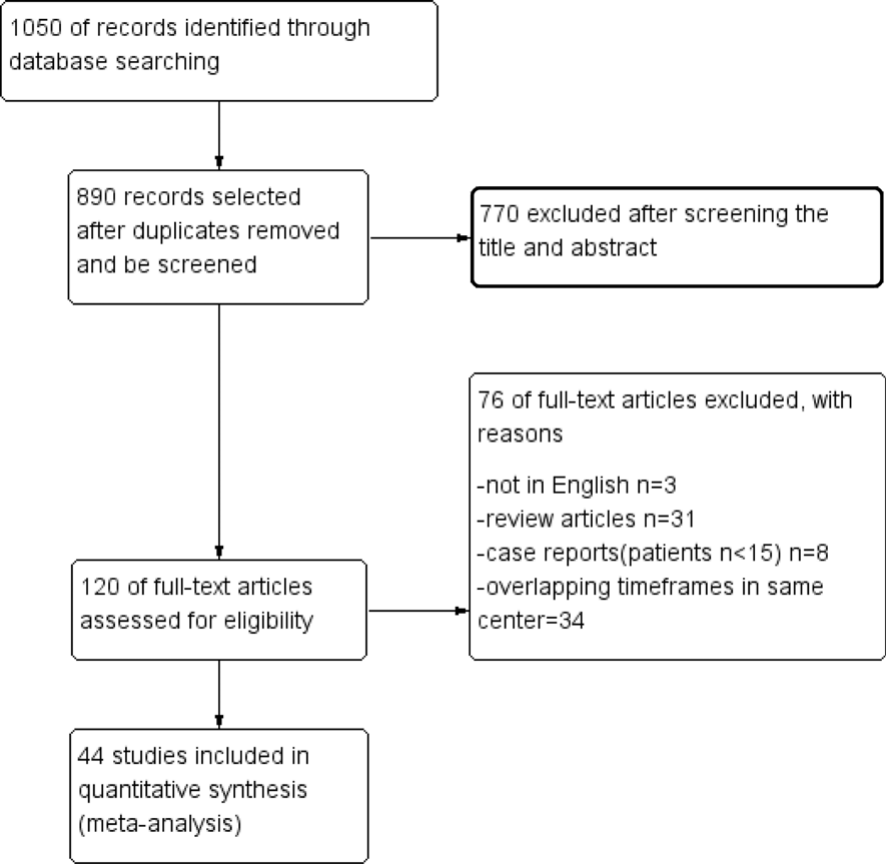
PRISMA flowchart.

The selection process is shown in detail in the PRISMA flowchart (**Figure 1**). According to a previous search strategy, 1050 results were initially identified from the online databases. After removing duplicates, 890 records remained. Then, 770 records were excluded after screening the titles and abstracts. Then, 76 reports were removed for various reasons, of which 34 were excluded because of overlapping timeframes in the same center. Finally, 44 studies were included in this meta-analysis (14–57). The quality assessment is shown in the **Supplementary Material**.

### Study Characteristics

The characteristics of the cohorts in the included studies were summarized in **Tables 1** and **2**. Overall, 44 studies involving 3730 patients were included. 1 was Randomized Controlled Trial, 5 were prospective studies, and 38 were retrospective studies. There are 2927 patients in 33 cohorts for CRT group; 614 patients in 13 cohorts in SBRT group; 189 patients in 6 cohorts in PRT group. The median age of the patients was 56 years (range, 47-73 years) in the overall studies, 56 for CRT group, 55.9 for SBRT group, 64.85 for PRT group. The median lesion size was 8 cm (range, 2.5–13.8 cm). The median percentage of previous-treatment patients was 75.6% (range 36.8%-100%) in the overall studies, 78.8% for CRT group, 79.2 for SBRT group, 67.8% for PRT group. The median percentage of patients with a class of no less than B was 20.23% (range 0%-41.18%) in the overall studies, 30.5% for CRT group, 13.89% for SBRT group, 37.25% for PRT group. The median dose was 48 Gy in the overall studies, 50 Gy for CRT group, 41 Gy for SBRT group, 72.6 GyE for PRT group. GyE is equal to the RBE multiplication with Gy; RBE of proton beam is 1.1; RBE of carbon ion is 3.

**Table 1:** Characteristics of the included studies.

Study	Country	Study design	Period	Type for RT	Patients (n)	Age (median)	Men(%)	Size (median, cm)	Target	CTC ≥B (%)	Dose (median)	Prior treatment (%)
**Hou et al.**	China	R	2000-2009	CRT	181	51.17	93.9		T 111, TT 70	16.6	50 Gy	90.6
**Tan et al.**	China	R	2012-2019	CRT	26	<=65 19(73%) >65 7(27%)	85%		TT	22	58 Gy	
**Toya et al.**	Japan	R	1999-2005	CRT	38	67	84.2	4	T	23.7	40 Gy	78.9
**Igaki et al.**	Japan	R	1990-2006	CRT	18	70	88.9		T	55.6	50 Gy	83.33
**Tanaka et al.**	Japan	R	1999-2011	CRT	67	65.5 (mean)	79.1		T	25.4	48.8 Gy (mean)	65.7
**Okazaki et al.**	Japan	R	2007-2013	CRT	56	69.1	85.7		T	30.5	50Gy	96.4
**Iwamoto et al.**	Japan	R	2008-2016	CRT	80	68	82.5	7.3	T	42.5	45 Gy	
**Yu et al.**	Korea	R	1998-2008	CRT	281	54	88.6		TT	16	30-54 Gy	86.1
**Rim et al.**	Korea	R	2005-2011	CRT	45	50	88.8	5.4	T	37.8	61.2 Gy	93.3
**Bae et al.**	Korea	R	2007-2015	CRT	47	60	79		T	34	40-45 Gy	74
**Huang et al.**	Taiwan	R	1997-2005	CRT	326	56.7 (mean)	85.3		T		60 Gy	
**Yeh et al.**	Taiwan	R	2004-2009	CRT	106	57	80		T	21.7	52 Gy	
**Pao et al.**	Taiwan	R	2007-2018	CRT	42	63	69		TT	40.5	48.75 Gy	64.3
**Onishi et al.**	Japan	R	1997-2012	CRT+HAIC	33	63	91	7			50 Gy	
**Kodama et al.**	Japan	R	–	CRT+HAIC	36	68	89		T	19.4	39 Gy	
**Han et al.**	Korea	R	2011-2016	CRT+HAIC	152	56	90.1	8.8		15.8	–	36.8
**Tang et al.**	China	R	2006-2008	CRT+TACE	185	49.7	83.8	9.49		8.6	40 Gy	
**Lu et al.**	China	R	2008-2011	CRT+TACE	30	58.5	70		T	33.3	40–52.5 Gy	
**Yamada et al.**	Japan	P	1998-2001	CRT+TACE	19	65.4 (mean)	78.9	5.2 (mean)	T	31.6	60 Gy	
**Shirai et al.**	Japan	R	2005-2008	CRT+TACE	19	64.8 (mean)	73.7	10.1	TT	31.6	45 Gy	
**Yoon et al.**	Korea	R	2002-2008	CRT+TACE	412	52	88.1	9.5	T 343, TT 69	35.9	40 Gy	
**Yoon et al.**	Korea	RCT	2013-2016	CRT+TACE	45	55	84.4	9.8	T		45 Gy	
**Yu et al.**	Korea	P, P II	2013-2016	CRT+TACE+hyperthermia	69	56	87	7.2	TT	8.7	47.25 GyE	
**Sugahara et al.**	Japan	R	1991-2005	PRT	35	63	80	6	TT	20	72.6 GyE	60
**Hashimoto et al.**	Japan	R	2013-2017	PRT	34	68	79.4			41.2	81.3 GyE	
**Komatsu et al.**	Japan	R	2001-2016	PRT	31	66.7	83.9	8.3		45.2	52.8-76 GyE	
**Sekino et al.**	Japan	R	2005-2014	PRT	21	73	80.9	8	TT	42.9	72.6 Gy	57.1
**Lee et al.**	Korea	R	2008-2011	PRT	27	55	81.5	7	TT	33.3	55 GyE	77.8
**Kim et al.**	Korea	R	2012-2015	PRT	41	55	85.4	5.8	TT	7.3	HCC 50 Gy, TVT 30 Gy	75.6
**Xi et al.**	China	R	2010-2012	SBRT	41	54	90.2	2.5	T		36 Gy	–
**Shui et al.**	China	R	2015-2017	SBRT	70	53.8	84.3		T	35.7	40Gy	
**Lou et al.**	China	R, multi-center	2008-2016	SBRT	75	53	85		TT	12	38Gy	–
**Dutta et al.**	India	P, P II	2017-2020	SBRT	72	63	96		TT	14	37.6 Gy	
**Kumar et al.**	India	R	2018-2020	SBRT	29	56	83	8.6		4	48 Gy	100
**Wang et al.**	Taiwan	P	2012	SBRT	20	68.55	60		TT	10	50 Gy	
**Choi et al.**	Korea	R	2010-2016	SBRT	24	56	83.3		T	12.5	45 Gy	79.2
**Hou et al.**	China	R	2011-2014	CRT	64	54.27	90.6	8.55	TT	21.9	54 Gy	
				CRT	54	54.37	79.6	7.5	TT	14.8	60 Gy	
**Zhao et al.**	China	R	2015-2018	CRT+TACE+Sorafenib	28	55.5	96.4	7.4	TT		–	
				CRT+TACE	35	54	91.4	6.6	TT		–	
**Li et al.**	China	R	2000-2017	CRT	154	47	87.7	9	TT	10.1	51 Gy	
				SBRT	133	51	90.2	8.1	TT	13.5	42 Gy	
**Nomura et al.**	Japan	R	2009-2017	CRT+HAIC	18	68 (mean)	83.3		T	61.1	50 Gy	
				CRT+HAIC+Sorafenib	14	68.5 (mean)	100		T	35.7	50 Gy	
**Lin et al.**	Taiwan	P	2002-2004	SBRT	22	59.5 (mean)	77.3	6.5	T		45Gy	
				CRT	21	54 (mean)	80.1	13.8	T		45Gy	
**Yang et al.**	Taiwan	R	2007-2016	SBRT	54	61 (mean)	77.8		T	35.2	45 Gy	55.6
				CRT	86	59.6 (mean)	75.6		T	50	51.5 Gy	38.4
**Que et al.**	Taiwan	R	2009-2016	SBRT+Sorafenib	18	55.39 (mean)	77.78		T	16.67	40 Gy	
				SBRT	36	59.83 (mean)	80.56			13.89	40 Gy	
**Khorprasert et al.**	Thailand	R	2007-2019	CRT	140	61.5	–	8.5	TT	31.65	45.8 Gy (mean)	68.1
				SBRT	20	55.9	–	3.9	TT	20	75.9 Gy (mean)	

RT, radiotherapy; CPC, Child–Pugh Class; R, retrospective; P, prospective; P II, phase II trial; RCT, Randomized Controlled Trial; PVT, CRT, conventional radiation therapy; SBRT, stereotactic body radiotherapy; PRT, particle radiotherapy; TT, thrombus and tumor; T, thrombus only; SR, Surgical resection; TACE, transarterial chemoembolization; HAIC, hepatic arterial infusion chemotherapy; GyE = RBE×Gy; RBE of proton beam is 1.1; RBE of carbon ion is 3.

**Table 2:** Baseline characteristics of CRT, SBRT and PRT cohorts.

	CRT cohorts	SBRT cohorts	PRT cohorts
**Cohorts (n)**	33	13	6
**Patients (n)**	2927	614	189
**Median age (median, years)**	56	55.9	64.85
**Men (median, %)**	85.15	83.15	81.2
**Median Child-Pugh≥B class (%)**	30.5	13.89	37.25
**Median radiation dose (GyE = RBE×Gy)**	50 Gy	41 Gy	72.6 GyE
**Prior treatment (median, %)**	78.9	79.2	67.8

GyE = RBE×Gy; RBE of proton beam is 1.1; RBE of carbon ion is 3.

### Effectiveness Outcomes

A total of 52 cohorts in 44 studies were included in the data synthesis. All valid data were extracted and are displayed in **Table 3**. Pooled data shown in **Table 4** and in Supporting Information. The 1-year pooled OS for CRT, SBRT, PRT were 45.3% (n = 2669, study = 30), 44.9% (n = 592, study = 12), 60.9% (n = 189, study = 6), respectively. The 2-year OS for CRT, SBRT, PRT were 20.4% (n = 2624, study = 29), 19.2% (n = 432, study = 8), 38.5% (n = 155, study = 5), respectively. Except pooled 1-year OS for SBRT group, PRT group; 2-year OS for PRT group with low heterogeneity, other pooled rates with high heterogeneity, respectively. The PRT group showed significantly higher than the CRT group and the SBRT group in OS (PRT vs. CRT: *p* = 0.005 for 1-year OS, *p* = 0.001 for 2-year OS; PRT vs. SBRT: *p* = 0.002 for 1-year OS, *p* = 0.004 for 2-year OS. Compared with previous meta-analysis, the results were stable for the CRT group and SBRT group as the increasing number of patients and studies (11).

**Table 3:** Clinical results.

Study	Follow up (month)	MST (months)	Type for RT	Patients (n)	1-year OS (%)	2-year OS (%)	Responser (n)	CR (%)	PR (%)	SD (%)	PD (%)	ORR (%)	LCR (%)
**Hou et al.**	10		CRT	181			181	29.3	31.5	33.7	5.5	60.8	94.5
**Tan et al.**	14.3	8	CRT	26	23	4	26	8	31	61		39	
**Toya et al.**		9.6	CRT	38	39.4	17.5	38	15.8	28.9	44.7	10.5	44.7	89.4
**Igaki et al.**		5.6	CRT	18	33.3	9	12	16.7	16.7	58.3	8.3	33.4	91.7
**Tanaka et al.**		9.4	CRT	67	39	9	67	7.5	37.3	23.9	31.3	44.8	68.7
**Okazaki et al.**	5.3	6.4	CRT	56			50	0	22	44	34	22	66
**Iwamoto et al.**		13.3	CRT	80	56	26.7							
**Yu et al.**	8	11.6	CRT	281	48.1	26.9	260	3.85	54.23	27.69	14.23	58.08	85.77
**Rim et al.**		13.9	CRT	45	51.5		45	6.7	55.6	31	6.7	62.3	93.3
**Bae et al.**	8		CRT	47	15	15	47	0	40	51	9	40	91
**Huang et al.**		3.8	CRT	326	16.7	5.5							
**Yeh et al.**	10	7	CRT	106	34.7	11	106	9.5	52	33	5.5	61.5	94.5
**Pao et al.**	4.4	6.6	CRT	42	30	19	27	14.8	59.3	25.9	0	74.1	100
**Onishi et al.**		12.4	CRT+HAIC	33	54.5	22	31	3.2	45.2	45.2	6.5	48.4	93.6
**Kodama et al.**		9.9	CRT+HAIC	36	47	20.3	36	8.3	41.7	50	0	50	100
**Han et al.**		13.5	CRT+HAIC	152	60	29.5	152	1.3	46.7	34.2	17.8	48	82.2
**Tang et al.**	10.7	12.3	CRT+TACE	185	51.6	28.4							
**Lu et al.**		13.02	CRT+TACE	30	62.4	20.81	30	16.7	53.3	20	10	70	90
**Yamada et al.**		7	CRT+TACE	19	40.6	10.2	19	0	57.9	42.1	0	57.9	100
**Shirai et al.**	9.4	10.3	CRT+TACE	19	47.4	23.7	19	0	36.8	52.6	10.5	36.8	89.4
**Yoon et al.**	10.6	10.6	CRT+TACE	412	42.5	22.8	409	6.6	33	46	14.4	39.6	85.6
**Yoon et al.**		12.8	CRT+TACE	45	53.3	26.8	45	0	28.9	51.1	20	28.9	80
**Yu et al.**	11.4		CRT+TACE +hyperthermia	69	85	62.9	69	34	36.2	15.3	14.5	70.2	85.5
**Sugahara et al.**	21	22	PRT	35	68	48	35	22.8	60	8.6	8.6	82.8	91.4
**Hashimoto et al.**	8.4		PRT	34	55		34	15	47	35	3	62	97
**Komatsu et al.**			PRT	31	47	24							
**Sekino et al.**	21		PRT	21	62	33							
**Lee et al.**	13.2	13.2	PRT	27	55.6	33.3	27	0	55.6	37	7.4	55.6	92.6
**Kim et al.**	15.2	34.4	PRT	41	73.2	51.1	41	34.2	48.8	14.3	2.4	83	97.3
**Xi et al.**	10	13	SBRT	41	50.3		41	36.6	39	17.1	7.3	75.6	92.7
**Shui et al.**	9.5	10	SBRT	70	40		62	9.7	69.4	6.4	14.5	79.1	85.5
**Lou et al.**		10	SBRT	75	38.7	13.3	75	22.7	73.3	4	0	96	100
**Dutta et al.**	6	11.4 (mean)	SBRT	72	38	10	54	0	36	42	22	36	78
**Kumar et al.**	8	15	SBRT	29	60		29	7	80	13		87	
**Wang et al.**	7.4	9.6 (mean)	SBRT	20	58		22	36.4	31.8	27.3	4.4	68.2	95.5
**Choi et al.**	8.4	20.8	SBRT	24	67.5	48.2	24	8.3	45.8	29.2	16.7	54.1	83.3
**Hou et al.**	11.8	10.46	CRT	64	35.8	16	64	1.6	51.6	12.5	34.3	53.2	65.7
		15.47	CRT	54	59.3	32	54	5.6	64.8	9.3	20.3	70.4	79.7
**Zhao et al.**	13	19	CRT+TACE+Sorafenib	28	72.4	48	28	10.7	35.7	28.6	25	46.4	75
	14.1	15.2	CRT+TACE	35	77.5	16	35	0	45.7	31.4	22.9	45.7	77.1
**Li et al.**	31	10	CRT	154	48.1	25.1							
		10	SBRT	133	46.5	29.3							
**Nomura et al.**		6.7	CRT+HAIC	18	21	6	32	9.4	50	21.9	18.7	59.4	81.3
		49.2	CRT+HAIC+Sorafenib	14	75	50							
**Lin et al.**		6	SBRT	22			14	7	71	21	0	78	100
		6.7	CRT	21									
**Yang et al.**		10.9	SBRT	54	34.9	15.3	45	11.1	51.1	33.33	4.4	62.2	95.53
		4.7	CRT	86	15.7	8	59	8.5	25.4	45.8	20.3	33.9	79.7
**Que et al.**	13.22 (mean)	12.5	SBRT+Sorafenib	18	55.6	17.7	18	33.33	44.44	11.11	11.11	77.77	88.88
	15.33 (mean)	7	SBRT	36	33.3	11.1	36	25	50	2.78	22.22	75	77.78
**Khorprasert et al.**	8.2	7.9	CRT	140	39.1	16.5	119	18.5	55.5	8.4	17.6	74	82.4
		11.9	SBRT	20	45	22							

Red font means overall survival in 1st year; overall survival in 2nd year.

**Table 4:** Comparison of pooled outcomes among groups.

Groups	Cohorts (n)	Patients (n)	*p*, Heterogeneity	I^2^	Pooled rates (95% CI)	*p*	*p*	
						(among three groups)	(between two groups)	*p*, Egger’s test,
**1-year OS**								
**Overall**	48	3450	0	87.1	47.3 (42.3, 52.4)			0.04
**CRT**	30	2669	0	89.1	45.3 (38.6, 52.1)	Q*=11.006, p*=0.004	PRT vs CRT	0.25
							Q=8.060, *p*=0.005	
**SBRT**	12	592	0.098	36.6	44.9 (39.5, 50.3)		SBRT vs CRT	0.114
							Q=0.009, *p*=0.926	
**PRT**	6	189	0.254	22.9	60.9 (52.6, 68.9)		PRT vs SBRT Q=9.922, *p*=0.002	0.39
**CRT + TACE**	7	745	0.001	73	53.2 (44.2, 62.2)	Q=7.856, *p*= 0.020	CRT+TACE vs CRT+HAIC	0.165
							Q=0.351, *p*=0.554	
**CRT + HAIC**	4	239	0.0012	72.8	48.0 (33.4, 62.7)		CRT vs CRT+HAIC	0.128
							Q=1.970, *p*=0.160	
**CRTal**	16	1574	0	90.1	36.1 (28.2, 44.3)		CRT vs CRT+TACE	0.676
							Q=7.612, *p*=0.006	
**2-year OS**								
**Overall**	42	3211	0	84.7	21.9 (18.0, 26.1)			0.357
**CRT**	29	2624	0	81.6	20.4 (15.9, 25.2)	Q=11.412, *p*=0.003	PRT vs CRT	0.725
							Q=10.353, *p*=0.001	
**SBRT**	8	432	0.001	72.3	19.2 (11.9, 27.5)		SBRT vs CRT	0.991
							Q=0.055, *p*=0.814	
**PRT**	5	155	0.128	44.1	38.5 (28.2, 49.3)		PRT vs SBRT	0.224
							Q=8.318, *p*=0.004	
**CRT + TACE**	7	745	0.466	0	23.2 (20.1, 26.4)	Q=6.021, *p*=0.049	CRT+TACE vs CRT+HAIC	0.401
							Q=0.106, *p*=0.744	
**CRT + HAIC**	4	239	0.106	50.9	21.5 (13.1, 31.2)		CRT vs CRT+HAIC	0.044
							Q=1.483, *p*=0.223	
**CRTal**	15	1529	0	85.2	15.5 (10.7, 21.0)		CRT vs CRT+TACE	0.977
							Q=6.020, *p*=0.014	
**ORR**								
**Overall**	40	2617	0	87	58.1 (52.2, 63.8)			0.151
**CRT**	26	1941	0	78.1	50.4 (45.1, 55.7)	Q=14.277, *p*=0.001	PRT vs CRT	0.863
							Q=7.455, *p*=0.006	
**SBRT**	10	439	0	88	72.7 (58.8, 84.7)		SBRT vs CRT	0.609
							Q=8.424,*p*=0.004	
**PRT**	4	137	0.024	68.3	72.1 (57.6, 84.7)		PRT vs SBRT	0.171
							Q=0.003, *p*=0.956	
**CRT + TACE**	6	557	0.007	68.4	45.1 (34.4, 56.0)	Q=0.725, *p*=0.696	CRT+TACE vs CRT+HAIC	0.403
							Q=0.247, *p*=0.619	
**CRT + HAIC**	3	219	–	–	48.4 (41.7, 55.1)		CRT vs CRT+HAIC	–
							Q=0.218, *p*=0.641	
**CRTal**	14	1036	0	79.1	50.7 (43.4, 58.0)		CRT vs CRT+TACE	0.142
							Q=0.707, *p*=0.400	
**LCR**								
**Overall**	38	2562	0	77	88.6 (85.5, 91.4)			0.654
**CRT**	25	1915	0	77.7	86.8 (83.0, 90.3)	Q=7.257, *p*=0.027	PRT vs CRT	0.872
							Q=7.213, *p*=0.007	
**SBRT**	9	410	0	78.4	90.4 (82.4, 96.3)		SBRT vs CRT	0.623
							Q=0.645, *p*=0.422	
**PRT**	4	137	0.638	0	95.1 (90.4, 98.5)		PRT vs SBRT	0.444
							Q=1.410, *p*=0.235	
**CRT + TACE**	6	557	0.007	68.9	87.0 (80.7, 92.3)	Q=0.962, *p*=0.618	CRT+TACE vs CRT+HAIC	0.403
							Q=0.827, *p*=0.363	
**CRT + HAIC**	3	219	–	–	93.5 (77.9, 1.0)		CRT vs CRT+HAIC	–
							Q=0.949, *p*=0.330	
**CRTal**	13	1010	0	84.6	86.1 (79.6, 91.6)		CRT vs CRT+TACE	0.599
							Q=0.019, *p*=0.891	

CRTal, CRT alone group. Red font means 2-groups comparison in 1-year OS, 2-year OS, ORR, LCR. Subgroups for CRT, SBRT, and PRT; CRT+TACE, CRT+HAIC, and CRTal.

The ORR for CRT, SBRT, PRT were 50.4% (n = 1941, study = 26), 72.7% (n = 439, study = 10), 72.1% (n = 137, study = 4), respectively. The LCR for CRT, SBRT, PRT were 86.8% (n = 1915, study = 25), 90.4% (n = 410, study = 9), 95.1% (n = 137, study = 4), respectively. Except pooled LCR for PRT group; other 5 pooled rates with high heterogeneity. The CRT group showed significantly lower than the PRT group (*p* = 0.006) and SBRT group (*p* = 0.004) in ORR. There was no statistical significance between PRT group and SBRT group in ORR (*p* = 0.956). The PRT group showed significantly higher than the CRT group (*p* = 0.007) in LCR.

In recent years, several studies have shown advantage in the combination of RT. We further compared the effects between CRT + TACE, CRT + HAIC, and CRTal groups (CRTal represents CRT alone). CRT + CATE group showed statistically significant advantage in survival prolongation than the CRT alone group (*p* = 0.006 for 1-year OS; *p* = 0.014 for 2-year OS). Pooled ORR and LCR was not statistically significant between three groups. Except pooled 2-year OS for CRT+TACE group; other pooled rates with high heterogeneity.

### Safety

Toxic effect events for groups showed in **Table 5**. For grade < 3 toxicity, the most common type of toxicity in CRT group was hepatotoxicity (977 events in 1007 patients), in SBRT group was hepatotoxicity as well (152 events in 139 patients), in PRT group was dermatological toxicity (44 events in 56 patients). For grade ≥ 3, the most frequent type of toxicity in CRT, SBRT, PRT group was hematological toxicity, hepatotoxicity, dermatological toxicity, respectively. PRT group showed advantage in avoiding hepatotoxicity than SBRT group (*p* = 0.003) and CRT group (*p* = 0.000); in avoiding hematological toxicity than CRT group (*p* = 0.003). There were no statistical difference among three groups in gastrointestinal toxicity (*p* = 0.112) and dermatological toxicity (*p* = 0.183). Five studies definitively reported late toxic events with total of 27 cases, 16 about gastrointestinal toxicity, 11 for dermatological toxicity.

**Table 5:** Comparison of toxic effect events for groups.

	Cohorts	Events	Total	Cohorts	Events	Total	Events rate (95%CI)	I ^2^	*p*	*p*
		for <grade 3			for ≥grade 3				(among three groups)	(among two groups)
**Hepatotoxicity**										
**CRT**	11	997	1007	13	178	1303	12.1 (6.8, 18.6)	87.7		PRT vs CRT
										Q=13.059, *p*=0.000
**SBRT**	5	152	139	6	40	209	14.7 (4.8, 28.1)	80.2	Q=16.0.39*, p*=0	SBRT vs CRT
										Q=0.198, *p*=0.656
**PRT**	2	7	68	4	2	127	6 (0, 3.8)	11		PRT vs SBRT
										Q=8.605, *p*=0.003
**Hematological**										
**CRT**	11	650	774	12	171	658	17.6 (7.8, 30.3)	92.7		PRT vs CRT
										Q=8.58, *p*=0.003
**SBRT**	3	87	95	4	18	165	10.8 (11.2, 28.6)	88.3	Q=8.97, *p*=0.011	SBRT vs CRT
										Q=0.50, *p*=0.482
**PRT**	3	31	103	4	3	128	2.2 (0.1, 6.9)	45.5		PRT vs SBRT
										Q=1.996, *p*=0.158
**Gastrointestinal**										
**CRT**	20	879	1951	17	62	1529	2.8 (0.6, 6.1)	85.8		PRT vs CRT
										Q=3.654, *p*=0.056
**SBRT**	7	141	268	6	1	193	0.1 (0, 1.8)	–	Q=4.374, *p*= 0.112	SBRT vs CRT
										Q=2.687, *p*=0.101
**PRT**	1	3	35	4	0	128	0	–		PRT vs SBRT
										Q=0.201, *p*=0.654
**Dermatological**										
**CRT**	5	62	268	4	0	226	0	–		PRT vs SBRT
										Q=0.080, *p*=0.778
**SBRT**	8	2	54	2	2	54	0.15 (0, 7.8)	–	Q=3.396, *p*=0.183	PRT vs CRT
										Q=1.728, *p*=0.189
**PRT**	2	44	56	4	3	121	0.1 (0, 5.8)	–		SBRT vs CRT
										Q=2.375, *p*=0.123

Red font means 2-groups comparison in Hepatotoxicity, Hematological, Gastrointestinal, Dermatological. Subgroups for CRT, SBRT, and PRT.

### Publication Bias

Egger’s test showed publication biases as follows: 1-year OS in the CRT, SBRT PRT groups (*p* = 0.25, 0.114, 0.390, respectively); 2-year OS in the CRT, SBRT PRT groups (*p* = 0.725, 0.991, 0.224); ORR in the CRT, SBRT PRT groups (*p* = 0.863, 0.609, 0.171). LCR in the CRT, SBRT PRT groups (*p* = 0.872, 0.623, 0.444). 1-year OS in the CRT+TACE, CRT+HAIC, CRTal groups (*p* = 0.165, 0.128, 0.676); 2-year OS in the CRT+TACE, CRT+HAIC, CRTal groups (*p* = 0.401, 0.044, 0.977, respectively); ORR in the CRT+TACE, CRTal groups (*p* = 0.403, 0.142). LCR in the CRT+TACE, CRTal groups (*p* = 0.403, 0.599).

## Discussion

There are 44 studies about external beam radiotherapy for HCC with MVI included in our study. The results showed PRT yields survival prolongation compared with SBRT and CRT. Meanwhile, PRT and SBRT both provide a higher ORR than CRT. In addition, radiotherapy based combination therapies are beneficial to prolong the survival of patients, especially for RT combined with TACE.

In cases of microvascular tumor invasion, especially to the main portal vein, the prognosis is poor. The reasons are as follows: (1) an extensive intrahepatic metastatic spread may result from shedding of HCC cells along the portal vein thrombosis; (2) when the main portal vein is completely blocked, liver function continues to deteriorate leading to liver failure occurs; and (3) exacerbation of portal hypertension causes refractory ascites and bleeding in the esophagus (58). Such physiological changes not only reduce patient survival, but also limit the choice of treatment. TACE is one of the standard treatments for unresectable liver cancer, especially for BCLC stage B tumors. However, it is contraindicated for portal vein tumor thrombus because post-operative ischemia may cause liver failure. At present, sorafenib is one of the first choices for HCC with MVI (59), but it has a slow-acting effect and is unable effectively alleviate the metastasis of liver cancer cells induced by PVTT. Kim et al. (60) reported that the median duration of efficacy of sorafenib alone in PVTT for liver tumor was less than five months.

Due to the rapid thrombosis of HCC, immediate reduction of macrovascular is important for follow-up treatment of the primary tumor. In our study, radiotherapy achieved a high ORR in a short time, especially SBRT and PRT. EBRT is a promising treatment and can recanalize the portal vein in a short time, improve nutrient supply to the liver, delay liver decompensation, and even reduce the Child–Pugh score, improving the survival rate. In addition, radiotherapy has a synergistic effect with mainstream treatments for HCC. TACE plus RT is an effective combination treatments. Radiotherapy targets vascular invasion and re-opens the portal vein, to facilitate conditions for TACE treatment. TACE can effectively inhibit the intrahepatic primary tumor and prevent recurrence of MVI. In our study, the CRT plus TACE group and the CRT plus HAIC group are superior to CRT group in survival (1-year OS: 53.2%, 48.0% vs 36.1%, *p* = 0.020; 2-year OS: 23.2%, 21.5% vs 15.5%, *p* = 0.049). Sorafenib, an inhibitor of RAF kinase and VEGFR, can limit tumor cell proliferation and tumor angiogenesis, decrease radiation-activated NF-κB and increase radiation-induced apoptosis (61–63). RT plus sorafenib displayed clinical benefit and safety for patients with macrovascular invasion (23, 27). A meta-analysis showed concurrent Sorafenib and RT significantly greater benefit in OS than did the non-concurrent treatment, and they recommend vascular tumor involvement as the only target of EBRT to avoid excessive toxicities (64). It illustrated the potential of radiotherapy in combination therapy.

Hepatocellular carcinoma (HCC) is a radiosensitive tumor with a dose-response relationship (65). Some large clinical studies showed that a high cumulative and per fraction dose can significantly improve the response rate, local control rate, and prolong survival in patients with HCC (66, 67). Dose of 40 to 45 Gy in 3 fractions or 40 to 50 Gy in 5 fractions (53 to 84 GyE) have been demonstrated to be safe with good therapeutic effect (65). Recently, conformal radiotherapy technique is converting from 3D to IMRT, which can improve curative effect. IMRT achieved higher biologically effective dose within fewer fractions and a shorter duration of therapeutic method than 3D-CRT. Compared with 3D-CRT, IMRT provides a survival benefit in HCC with MVI (29). Meanwhile, a study showed median OS and LCR in the IMRT group were similar to those of the SBRT group for HCC with MVI (47). However, study about IMRT for HCC with MVI is scarce, and the clinical efficacy requires more clinical data to support. SBRT and PRT have a dose advantage over conformal radiotherapy by delivering large doses of radiation to the target tumor volume in a small fraction. The treatments can be completed in a short time because of a higher biologically effective dose. A short course of treatment is conducive due to less interference with other therapeutic methods, reducing toxicity. The outcomes in our study are consistent with prevailing views about the dose-response. SBRT and PRT are associated with higher response rates than CRT. PRT show higher survival rates than CRT.

SBRT has made excellent progress in the field of radiation therapy. However, due to the inherent physical characteristics of photons, SBRT has limited advantage with respect to side effects and liver toxicity. Based on the findings, SBRT is inferior to PRT in avoiding hepatotoxicity. Due to its excellent physical properties, PRT can significantly reduce dose exposure to normal tissues when high doses are used to treat target tumors. PRT is expected to be an ideal treatment for HCCs with high Child-Pugh score. The dosimetric superiority of PRT was correlated with the tumor location. A study by Gandhi et al. showed that PRT can reduce radiation toxicity to target tumor located in the dome and of a size >3 cm (68). Some clinical studies have also proven the safety and efficiency of PRT in the treatment of inferior vena cava tumor thrombi (39, 40). In our study, PRT showed an advantage over SBRT and CRT with respect to hepatotoxicity and hematological toxicity in ≥ grade 3 toxic effect events.

This meta-analysis has several limitations. On one side, meta-analysis is controversial for observational studies. It has been known that RCTs are the most effective means of reducing bias, and meta-analyses of RCTs provide the strongest evidence support (69). However, randomized controlled trial of radiation oncology is difficult to carry out. Radiation therapy competes with other treatments. 60% of all patients with cancer have received primarily treatments in other disciplines before receiving radiotherapy (70). Results from RCTs cannot always be feasible to answer clinical questions, especially in oncology. Meta-analysis of observational studies is an effective method to overcome the information gaps resulting from the insufficient RCT-based data (71). Meta-analysis of observational studies with high-quality did not show significantly different effect sizes from those of RCTs (72). On the other side, heterogeneity is inevitable because of the integrated information in studies with the diversities of designs and populations. The radiotherapy standard of HCC with MVI has not reached a consensus. Too strict inclusion criteria can reduce heterogeneity among studies, but cannot help to address clinical challenges in the real world. Heterogeneity should not be seen as an obstacle to the conclusion. Heterogeneity in meta-analysis requires statistical evaluation and interpretation of clinical phenomena to guide clinical decision-making and solve real-world problems (73).

## Conclusion

When compared with SBRT and CRT groups, PRT can prolong survival and reduces the occurrence of hepatotoxic events in patients with HCC and MVI. PRT and SBRT have advantages over CRT with respect to the ORR. A combination treatment based on radiotherapy can provide survival benefits to these patients. Since some of the included studies were observational studies, high-quality comparative studies are needed to provide reliable conclusions.

## Data Availability Statement

The original contributions presented in the study are included in the article/**Supplementary Material**. Further inquiries can be directed to the corresponding authors.

## Author Contributions

GW designed the study, acquired data, analyzed data, drafted the manuscript and accepted final version. GH designed the study, acquired the data, reviewed the manuscript and accepted final version. JH acquired the data, reviewed the manuscript and accepted final version. LL designed the study, reviewed the manuscript and accepted final version. SP designed the study, reviewed the manuscript and accepted final version. YL designed the study, analyzed data, reviewed the manuscript and accepted final version. WZ designed the study, analyzed data, reviewed the manuscript and accepted final version. All authors contributed to the article and approved the submitted version.

## Funding

This study is supported by the National Key Research and Development Program of China (2017YFA0205200), the National Natural Science Foundation of China (81901857, 81771957), Guangdong Provincial Key Laboratory of Tumor Interventional Diagnosis and Treatment (2021B1212040004), and the Science and Technology Development Fund, Macau SAR (0011/2019/AKP).

## Conflict of Interest

The authors declare that the research was conducted in the absence of any commercial or financial relationships that could be construed as a potential conflict of interest.

## Publisher’s Note

All claims expressed in this article are solely those of the authors and do not necessarily represent those of their affiliated organizations, or those of the publisher, the editors and the reviewers. Any product that may be evaluated in this article, or claim that may be made by its manufacturer, is not guaranteed or endorsed by the publisher.
