# Supplementary material for: Comparison of External Beam Radiation Therapy Modalities for Hepatocellular Carcinoma With Macrovascular Invasion: A Meta-Analysis and Systematic Review
Source: Front Oncol. 2022 Feb 15;12:829708. doi: 10.3389/fonc.2022.829708 (PMC8887617; doi:10.3389/fonc.2022.829708)
Supplement: Supplementary file 1 [file DataSheet_1.docx]

# Supplementary Material

# Search strategy

Web of science

| **Search number** | **Query** | **Results** |
| --- | --- | --- |
| #1 | TS=((hepatocellular OR liver OR hepatocellular) AND (cancer OR tumor OR tumour OR carcinoma )) | 312,157 |
| #2 | TS=(((proton therapy) OR proton OR particle OR (heavy ion) OR carbon) AND (beam irradiation OR radiation OR irradiation OR beam radiation OR radiotherapy OR beam therapy OR therapeutic)) | 219,415 |
| #3 | TS=((((Intensity modulated) OR (3 dimensional conformal) OR 3D OR stereotactic) AND (Radio*)) OR IMRT OR SBRT) | 84,898 |
| #4 | #2 OR #3 | 300,357 |
| #5 | TS=(Mortality OR (adverse effect) OR (adverse event) OR (side effect) OR (toxicit) OR disease free survival OR overall survival OR local control OR tumour control OR tumour control OR (treatment outcome) OR (outcome assessment) OR (survival rate)) | 3,453,412 |
| #6 | TS=(clinical trials OR observation studies OR prospective study OR retrospective study ) | 2,327,684 |
| #7 | TS=((vena or vein or venous or inferior or vascular) and thromb*) | 163,197 |
| #8 | #1 AND #4 AND #5 AND #6 AND #7 | 80 |

**ClinicalTrials**

hepatocellular carcinoma and (radiotherapy or Radiation therapy) results: 255

## Cochrane library

| **Search number** | **Query** | **Results** |
| --- | --- | --- |
| #1 | MeSH descriptor: [Hepatocellular Carcinoma] explode all trees | 1893 |
| #2 | MeSH descriptor: [Radiotherapy] explode all trees | 6456 |
| #3 | #1 and #2 | 52 |

**EMBASE**

| **Search number** | **Query** | **Results** |
| --- | --- | --- |
| #1 | ((hepatocellularc or liver or hepatobiliary) and (cancer or tumor or tumour or oncology or carcinoma)).mp. [mp=title, abstract, heading word, drug trade name, original title, device manufacturer, drug manufacturer, device trade name, keyword heading word, floating subheading word, candidate term word] | 526238 |
| #2 | exp proton therapy/ or exp heavy ion/ or exp particle therapy/ or exp ion therapy/ or exp heavy ion radiation/ OR (((proton therapy) OR proton or particle OR (heavy ion) OR (light ion) OR hadron OR carbon) AND (beam irradiation OR radiation OR irradiation OR beam radiation OR (radiotherapy) OR (beam therapy) OR therapeutic)) | 133466 |
| #3 | exp radiotherapy/ or exp hypofractionated radiotherapy/ or exp intensity modulated radiation therapy/ or exp stereotactic body radiation therapy/ or exp conformal radiotherapy/ | 579911 |
| #4 | exp vein thrombosis/ or exp liver vein thrombosis/ or exp portal vein thrombosis/ or exp thrombosis/ or (Thrombo* and (vena or vein or venous or inferior)).mp. [mp=title, abstract, heading word, drug trade name, original title, device manufacturer, drug manufacturer, device trade name, keyword heading word, floating subheading word, candidate term word] | 424964 |
| #5 | (Mortality or adverse effect or adverse event or side effect or toxicit or xerostomi or disease free survival or overall survival or local control or tumour control or tumour control).mp. or exp mortality/ or exp treatment outcome/ or exp survival rate/ or exp disease free survival/ [mp=title, abstract, heading word, drug trade name, original title, device manufacturer, drug manufacturer, device trade name, keyword heading word, floating subheading word, candidate term word] | 4601664 |
| #6 | (clinical trials or observation studies or prospective study or retrospective study).mp. [mp=title, abstract, heading word, drug trade name, original title, device manufacturer, drug manufacturer, device trade name, keyword heading word, floating subheading word, candidate term word] | 2316710 |
| #7 | 1 and (2 or 3) and 4 and 5 and 6 | 560 |

**MEDLINE**

| **Search number** | **Query** | **Results** |
| --- | --- | --- |
| #1 | ((hepatocellularc or liver or hepatobiliary) and (Neoplasm or cancer or tumor or tumour or malignant or oncology or carcinoma)).mp. or exp Liver Neoplasms/ or exp Carcinoma, Hepatocellular/ | 284681 |
| #2 | exp Thrombosis/ and ((exp Veins/ and vena.mp.) or vein.mp. or venous.mp. or inferior.mp. or vascular.mp.) [mp=title, abstract, original title, name of substance word, subject heading word, floating sub-heading word, keyword heading word, organism supplementary concept word, protocol supplementary concept word, rare disease supplementary concept word, unique identifier, synonyms] | 74077 |
| #3 | (Mortality or adverse effect or adverse event or side effect or toxicit or xerostomi or disease free survival or overall survival or local control or tumour control or tumour control).mp. or exp Mortality/ or exp Treatment Outcome/ or Outcome Assessment, Health Care/ or exp Survival Rate/ or exp Disease-Free Survival/ [mp=title, abstract, original title, name of substance word, subject heading word, floating sub-heading word, keyword heading word, organism supplementary concept word, protocol supplementary concept word, rare disease supplementary concept word, unique identifier, synonyms] | 2383050 |
| #4 | exp Radiotherapy/ or exp Radiotherapy, Intensity-Modulated/ or (Intensity modulated and radiotherapy).mp. or 3 dimensional conformal.mp. or (stereotactic and body).mp. or exp Radiotherapy Planning, Computer-Assisted/ [mp=title, abstract, original title, name of substance word, subject heading word, floating sub-heading word, keyword heading word, organism supplementary concept word, protocol supplementary concept word, rare disease supplementary concept word, unique identifier, synonyms] | 201647 |
| #5 | exp proton therapy/ or exp heavy ion/ or exp heavy ion radiation/ or ((proton therapy or proton or particle or heavy ion or light ion or hadron or carbon) and (beam irradiation or radiation or irradiation or beam radiation or radiotherapy or beam therapy or therapeutic)).mp. [mp=title, abstract, original title, name of substance word, subject heading word, floating sub-heading word, keyword heading word, organism supplementary concept word, protocol supplementary concept word, rare disease supplementary concept word, unique identifier, synonyms] | 85491 |
| #6 | 4 or 5 | 4 or 5 |
| #7 | 1 and 2 and 3 and 6 | 103 |

# Forest plots of outcomes


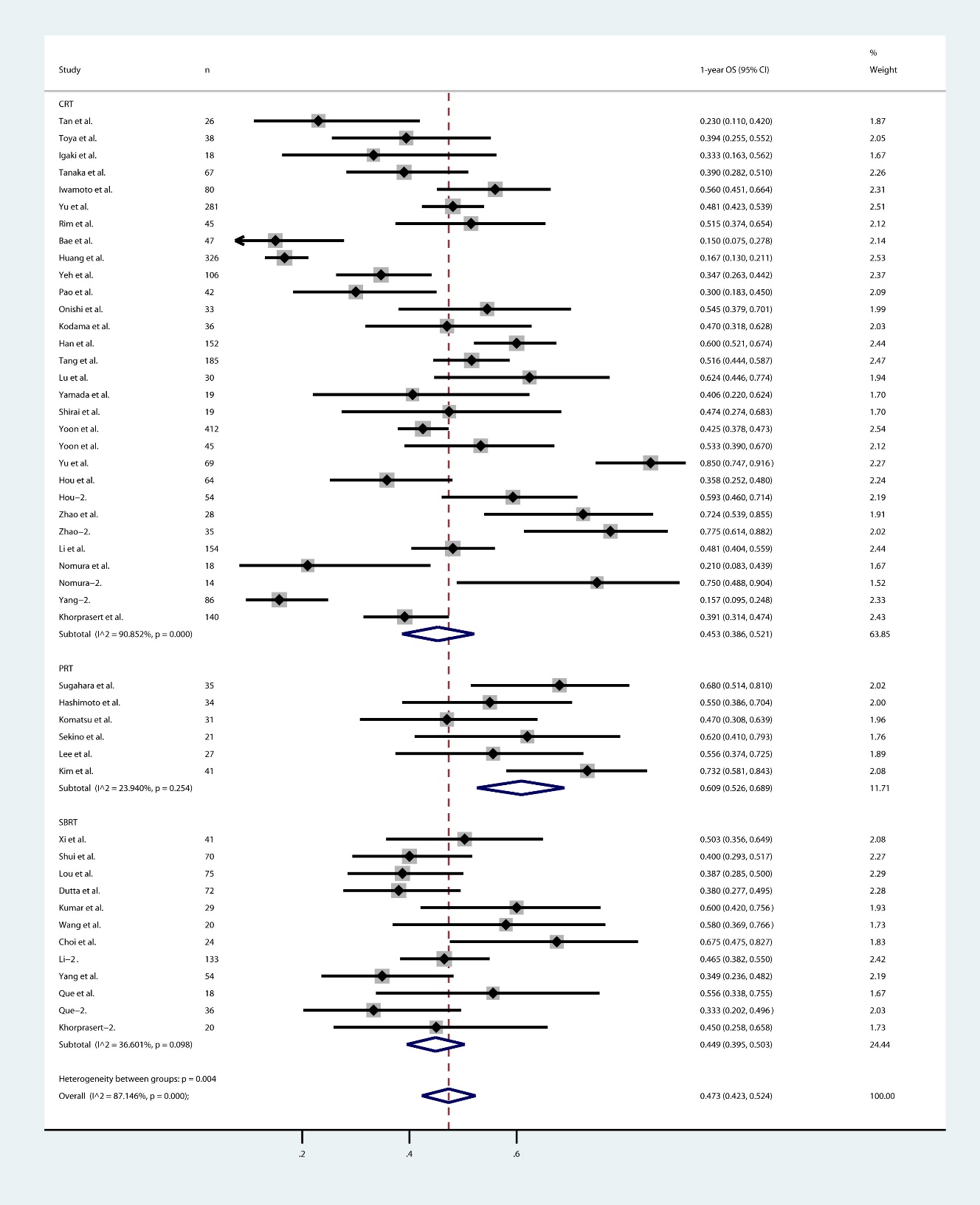


e-Fig.1 1-year overall survival for PRT, SBRT, CRT groups.


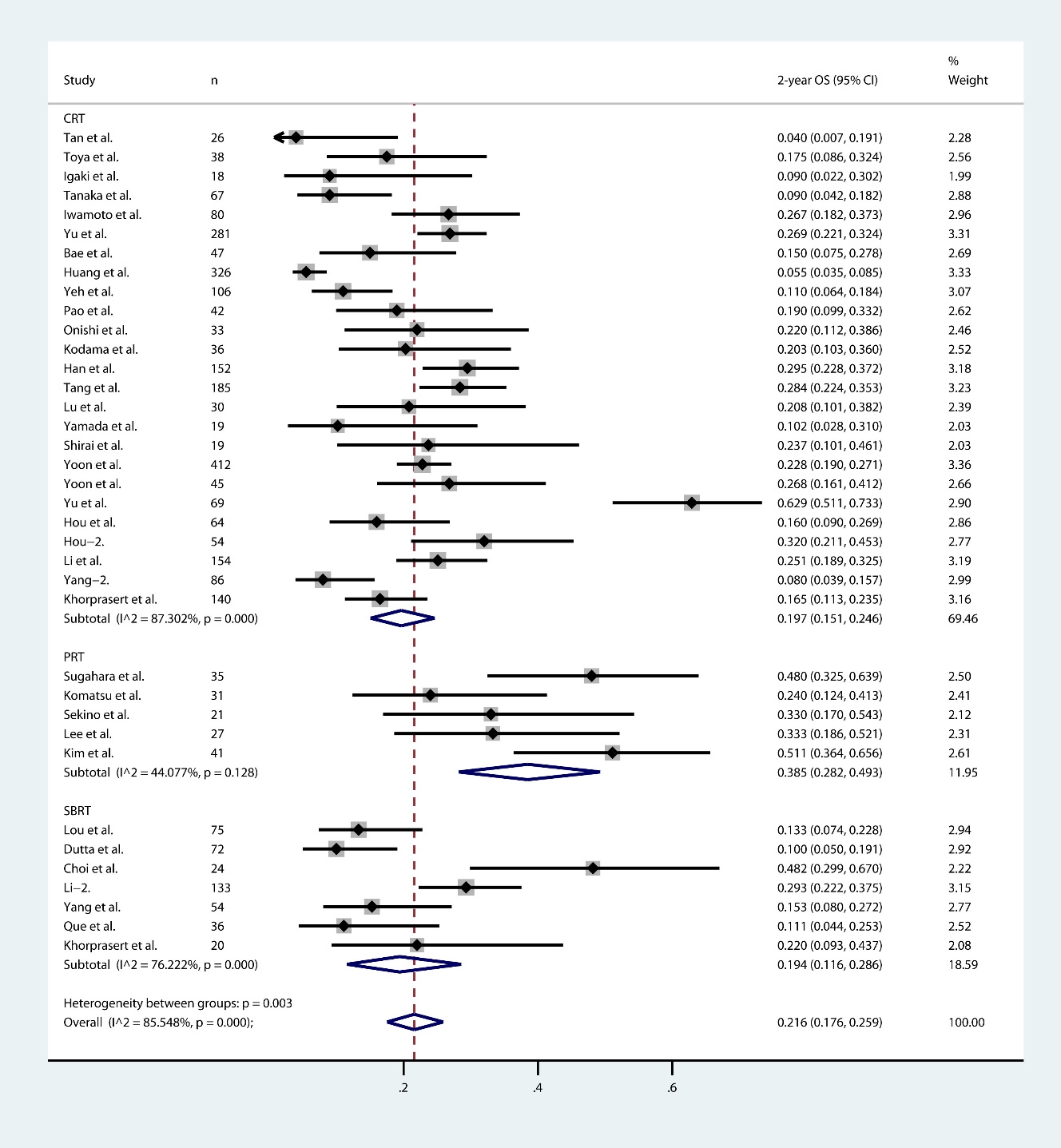


e-Fig.2 2-year overall survival for PRT, SBRT, CRT groups.


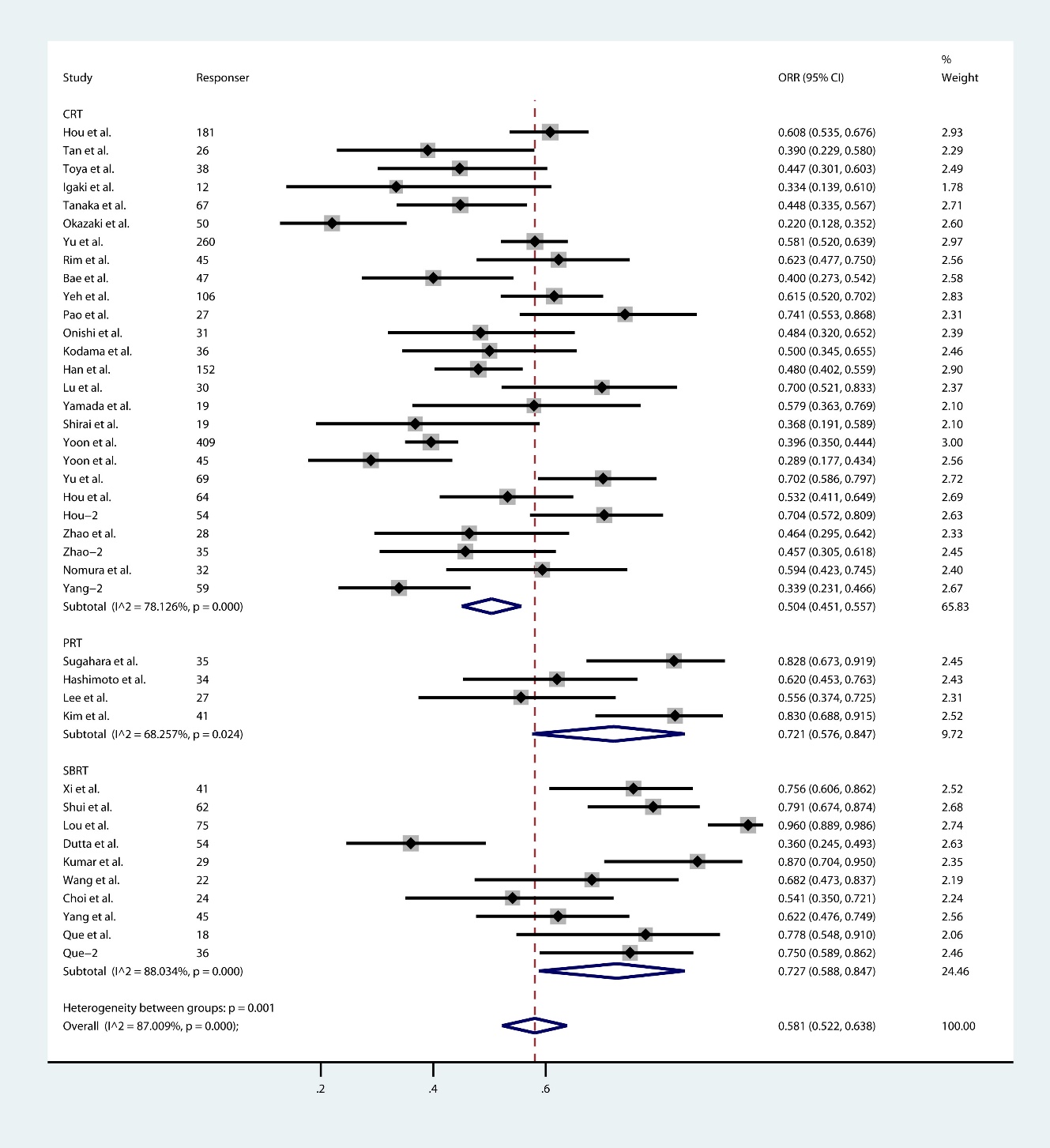


e-Fig.3 Objective response rate for PRT, SBRT, CRT groups.


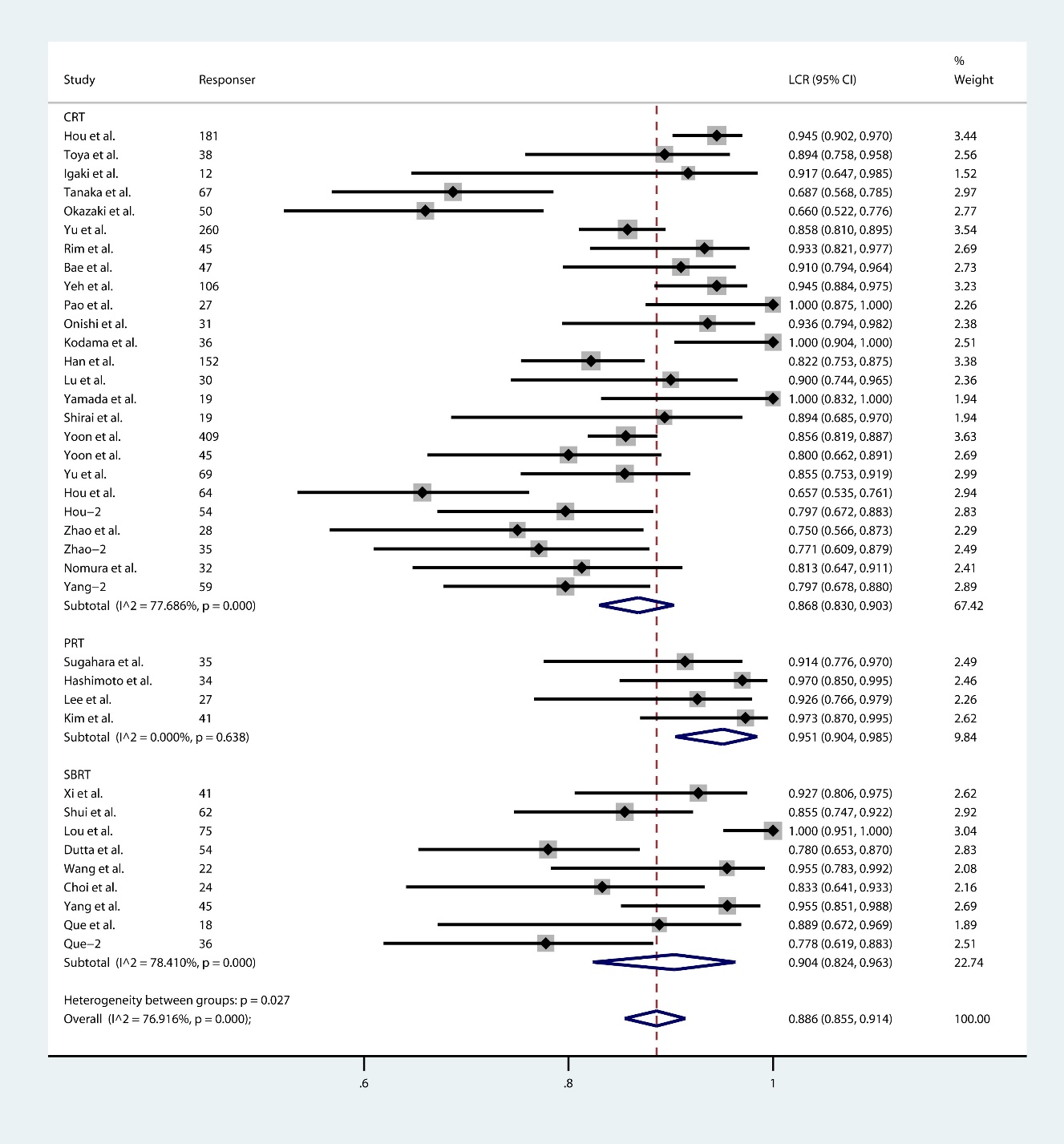


e-Fig.4 Local contral rate for PRT, SBRT, CRT groups.


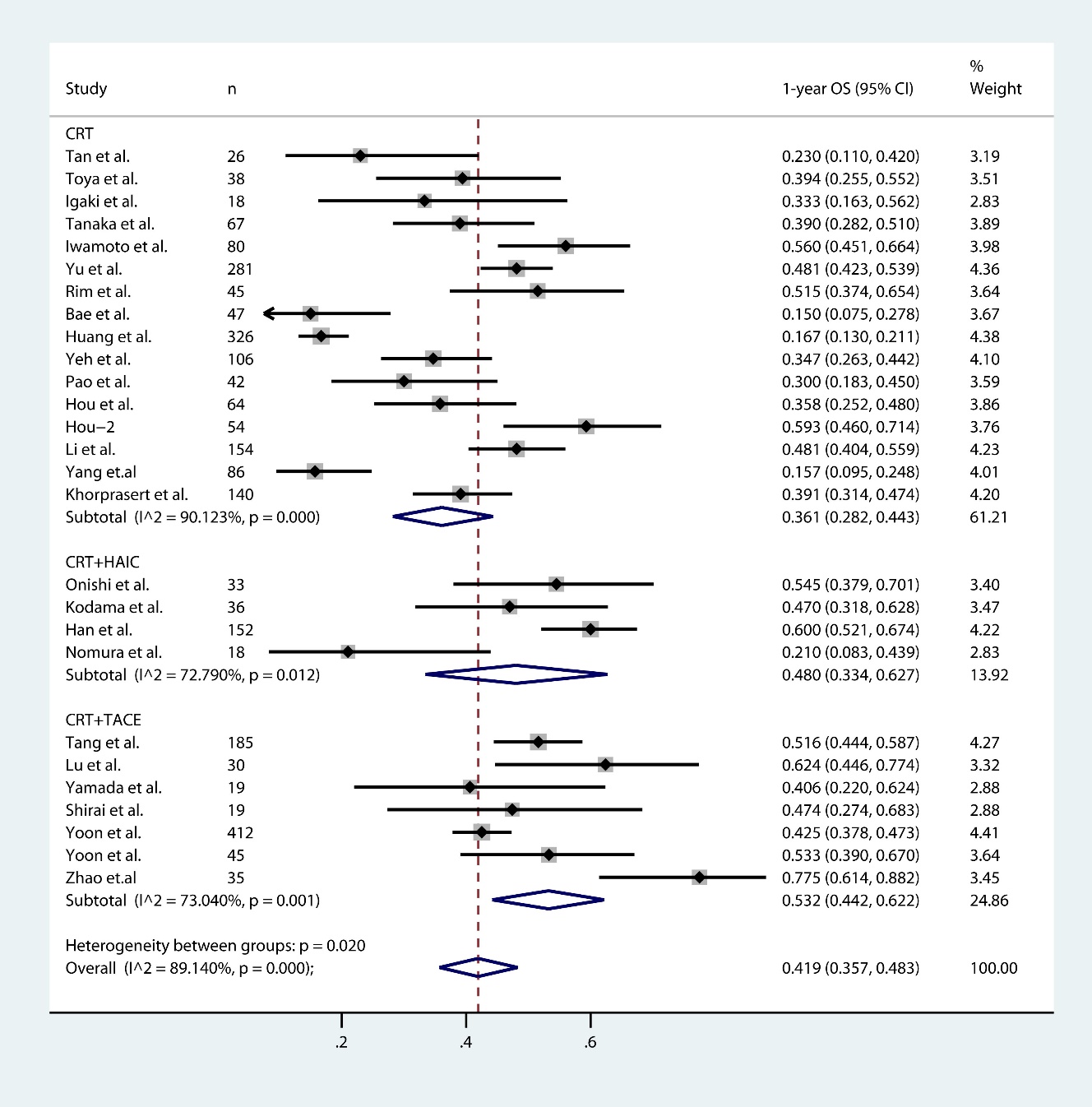


e-Fig.5 1-year overall survival for CRT+TACE, CRT+HAIC, CRT alone groups.


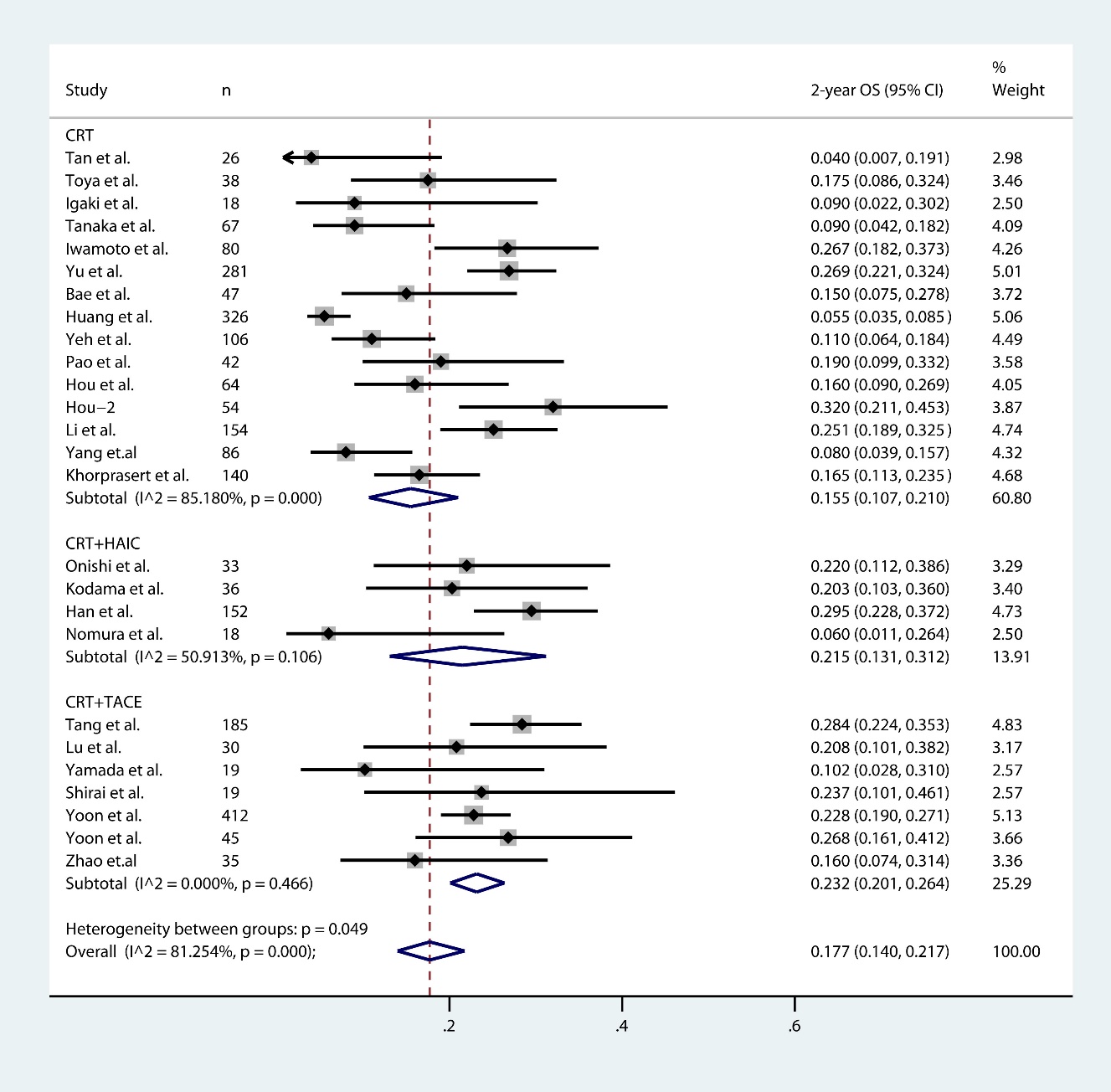


e-Fig.6 2-year overall survival for CRT+TACE, CRT+HAIC, CRT alone groups.


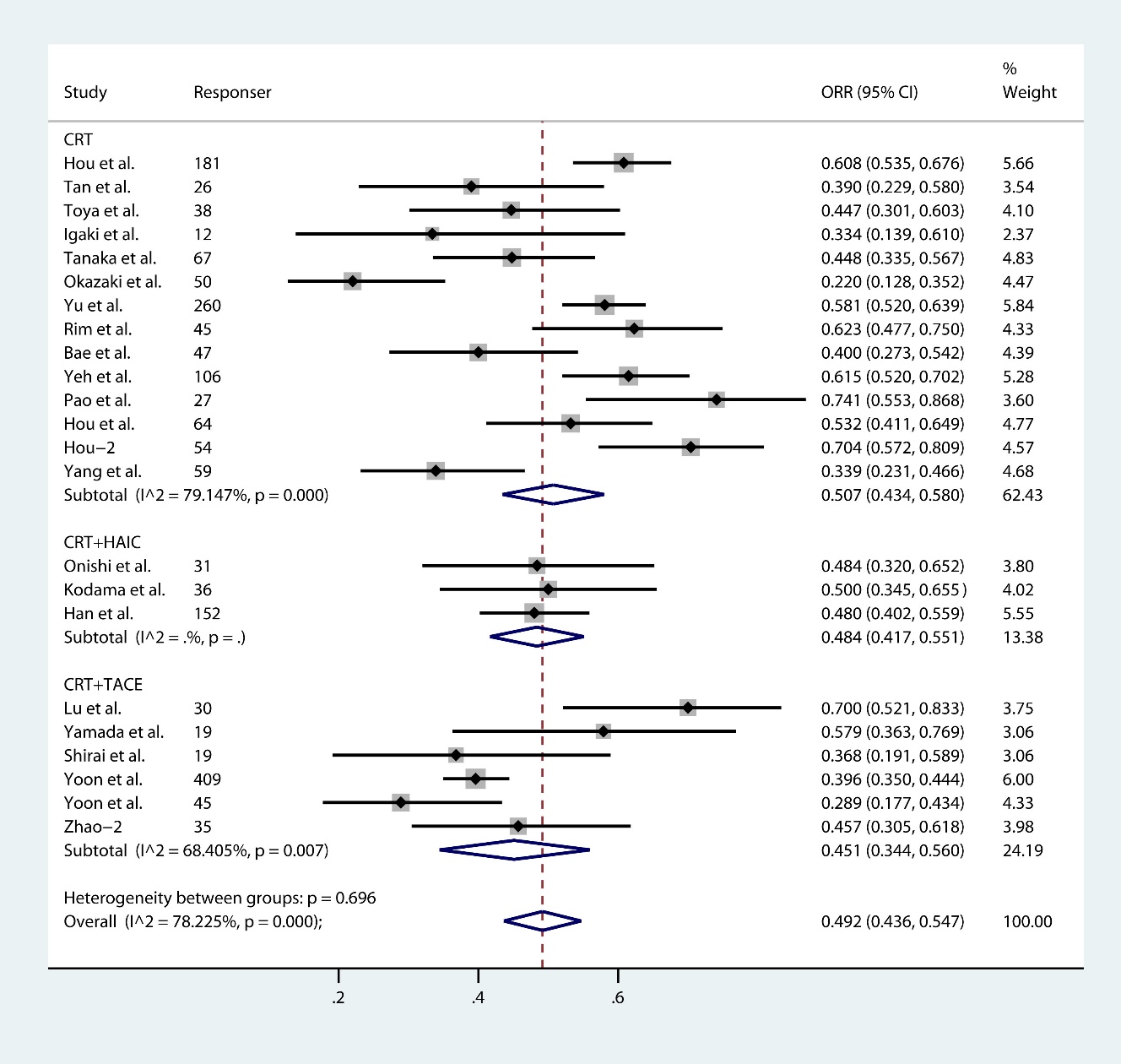


e-Fig.7 Objective response rate for CRT+TACE, CRT+HAIC, CRT alone groups.


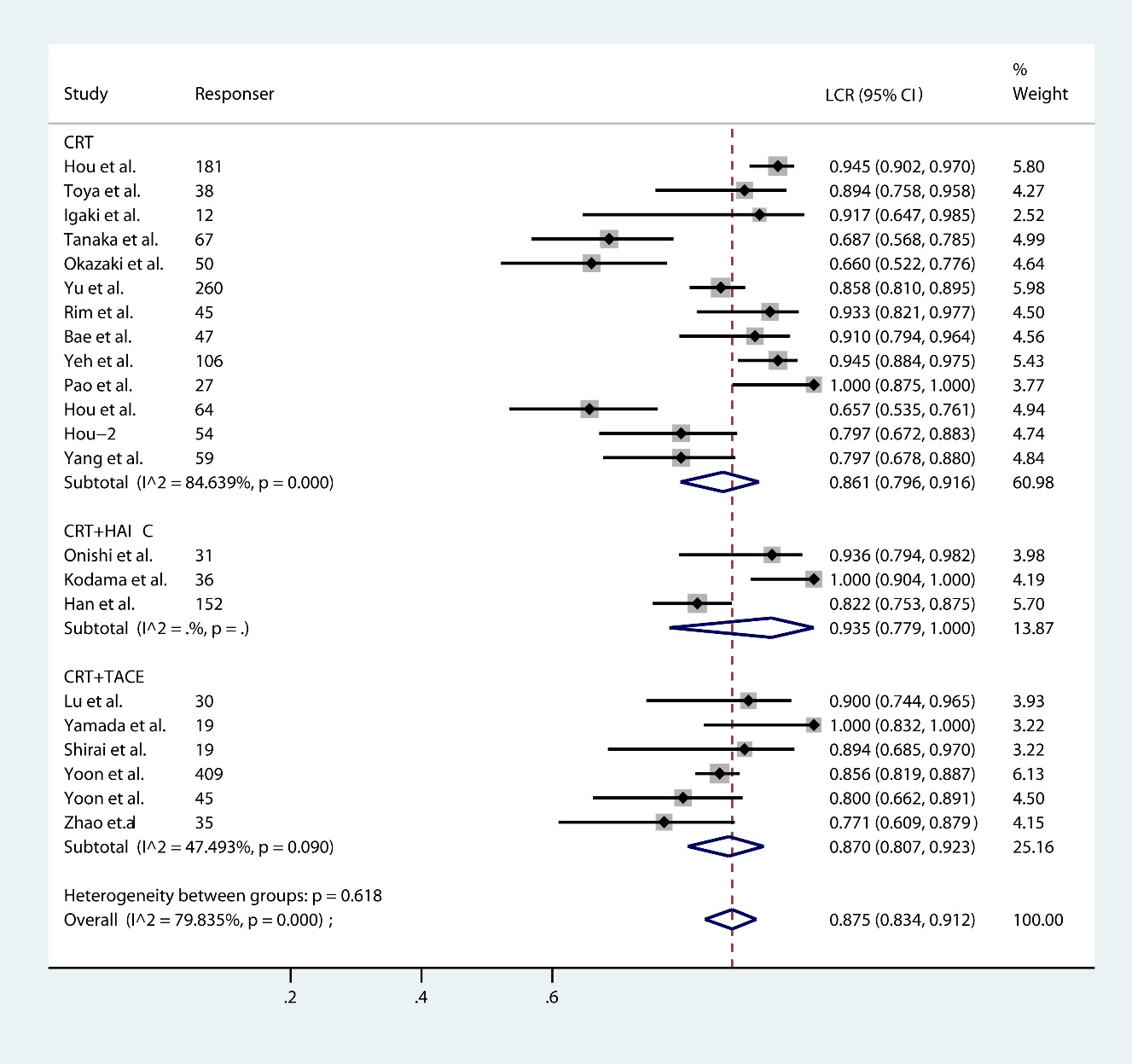


e-Fig.8 Local contral for CRT+TACE, CRT+HAIC, CRT alone groups.

3. study quality evaluation

e-Table. Evaluation of Included Studies Using Modified Newcastle-Ottawa Scale

| Author's name | Study population  clearly defined | Consecutive  patients included | Patients represent population  with HCC having MVI | Accurate description of  with HCC having MVI | Assessment of outcome  well defined | Toxicity  well defined | Follow-up long  enough for outcomes |
| --- | --- | --- | --- | --- | --- | --- | --- |
| Hou et al. | Yes | Yes | Yes | Yes | Yes | Yes | No |
| Tan et al. | Yes | Yes | Yes | Yes | Yes | Yes | Yes |
| Toya et al. | Yes | Yes | No | Yes | Yes | No | Yes |
| Igaki et al. | Yes | Yes | Yes | Yes | Yes | No | Yes |
| Tanaka et al. | Yes | Yes | Yes | Yes | Yes | No | Yes |
| Okazaki et al. | Yes | Yes | Yes | Yes | No | Yes | No |
| Iwamoto et al. | Yes | Yes | Yes | Yes | No | No | Yes |
| Yu et al. | Yes | Yes | Yes | Yes | Yes | Yes | Yes |
| Rim et al. | Yes | Yes | Yes | Yes | Yes | No | No |
| Bae et al. | Yes | Yes | Yes | Yes | Yes | No | Yes |
| Huang et al. | Yes | Yes | Yes | Yes | No | No | Yes |
| Yeh et al. | Yes | Yes | Yes | Yes | Yes | No | Yes |
| Pao et al. | Yes | Yes | Yes | Yes | Yes | Yes | Yes |
| Onishi et al. | Yes | Yes | Yes | Yes | Yes | Yes | Yes |
| Kodama et al. | Yes | Yes | Yes | Yes | Yes | Yes | Yes |
| Han et al. | Yes | Yes | Yes | Yes | Yes | Yes | Yes |
| Tang et al. | Yes | Yes | Yes | Yes | Yes | Yes | Yes |
| Lu et al. | Yes | Yes | Yes | Yes | Yes | No | Yes |
| Yamada et al. | Yes | Yes | Yes | Yes | Yes | Yes | Yes |
| Shirai et al. | Yes | Yes | No | Yes | Yes | Yes | Yes |
| Yoon et al. | Yes | Yes | Yes | Yes | Yes | Yes | Yes |
| Yoon et al. | Yes | Yes | Yes | Yes | Yes | Yes | Yes |
| Yu et al. | Yes | Yes | Yes | Yes | Yes | Yes | Yes |
| Sugahara et al. | Yes | Yes | Yes | Yes | Yes | Yes | No |
| Hashimoto et al. | Yes | Yes | Yes | Yes | No | No | No |
| Komatsu et al. | Yes | Yes | Yes | Yes | No | Yes | Yes |
| Sekino et al. | Yes | Yes | Yes | Yes | Yes | Yes | Yes |
| Lee et al. | Yes | Yes | Yes | Yes | Yes | No | Yes |
| Kim et al. | Yes | Yes | Yes | Yes | Yes | Yes | Yes |
| Xi et al. | Yes | Yes | Yes | Yes | Yes | Yes | No |
| Shui et al. | Yes | Yes | Yes | Yes | Yes | No | No |
| Lou et al. | Yes | Yes | Yes | Yes | Yes | No | Yes |
| Dutta et al. | Yes | Yes | Yes | Yes | Yes | No | Yes |
| Kumar et al. | Yes | Yes | Yes | Yes | Yes | No | No |
| Wang et al. | Yes | Yes | Yes | Yes | Yes | Yes | Yes |
| Choi et al. | Yes | Yes | Yes | Yes | Yes | Yes | Yes |
| Hou et al. | Yes | Yes | Yes | Yes | Yes | Yes | Yes |
| Zhao et al. | Yes | Yes | Yes | Yes | Yes | Yes | Yes |
| Li et al. | Yes | Yes | Yes | Yes | Yes | No | Yes |
| Nomura et al. | Yes | Yes | Yes | Yes | Yes | No | Yes |
| Lin et al. | Yes | Yes | Yes | Yes | No | Yes | No |
| Yang et al. | Yes | Yes | Yes | Yes | Yes | Yes | Yes |
| Que et al. | Yes | Yes | Yes | Yes | Yes | Yes | Yes |
| Khorprasert et al. | Yes | Yes | Yes | Yes | Yes | No | Yes |
